# The Effect of Fruit and Berry Pomaces on the Growth Dynamics of Microorganisms and Sensory Properties of Marinated Rainbow Trout

**DOI:** 10.3390/microorganisms11122960

**Published:** 2023-12-11

**Authors:** Mati Roasto, Mihkel Mäesaar, Tõnu Püssa, Dea Anton, Reelika Rätsep, Terje Elias, Salli Jortikka, Merilin Pärna, Karmen Kapp, Marek Tepper, Kristi Kerner, Kadrin Meremäe

**Affiliations:** 1 Chair of Veterinary Biomedicine and Food Hygiene, Institute of Veterinary Medicine and Animal Sciences, Estonian University of Life Sciences, Kreutzwaldi 56/3, 51006 Tartu, Estonia; mihkel.maesaar@emu.ee (M.M.); tonu.pyssa@emu.ee (T.P.); dea.anton@emu.ee (D.A.); terje.elias@emu.ee (T.E.); salli.jortikka@emu.ee (S.J.); kadrin.meremae@emu.ee (K.M.); 2 Polli Horticultural Research Centre, Chair of Horticulture, Institute of Agricultural and Environmental Sciences, Estonian University of Life Sciences, Uus 2, 69108 Polli, Estonia; reelika.ratsep@emu.ee; 3 Chair of Food Science and Technology, Institute of Veterinary Medicine and Animal Sciences, Estonian University of Life Sciences, Kreutzwaldi 56/5, 51006 Tartu, Estonia; merilin.parna@emu.ee (M.P.); marek.tepper@emu.ee (M.T.); kristi.kerner@emu.ee (K.K.); 4 Division of Pharmaceutical Biosciences, Faculty of Pharmacy, University of Helsinki, Viikinkaari 5E, P.O. Box 56, FI-00014 Helsinki, Finland; karmen.kapp@helsinki.fi

**Keywords:** rainbow trout, fruit and berry pomaces, marinades, microbial counts, *Listeria monocytogenes*, challenge test, sensory evaluation

## Abstract

Plant pomaces in suitable forms (powders, extracts) can be used in foods of animal origin to increase the nutritional value and safety of these foods. In the present study, water extracts of apple, black currant, rhubarb and tomato pomaces were used in fish marinade solutions to evaluate their effect on the growth dynamics of microorganisms and the growth potential of *Listeria monocytogenes* by challenge testing. The results showed that mesophilic aerobic microorganisms, *Pseudomonas* spp., yeasts and moulds remained at acceptable levels throughout the predetermined storage period. The challenge test results showed that the overall growth potential of *L*. *monocytogenes* in all marinated rainbow trout samples remained at ≤0.5 log_10_ cfu/g during the study period, and none of the marinated fish samples supported the growth of *L. monocytogenes*. In addition, the effect of fruit and berry pomaces on the sensory properties of marinated rainbow trout samples was evaluated. The results revealed that it is possible to effectively use fruit and berry pomaces in marinated fish products, ensuring food safety, high microbiological quality, acceptable sensory characteristics and a sufficiently long shelf life of the products.

## 1. Introduction

Improved management of by-products from food production is an essential step in the transition towards a circular and bio-economy [1]. The production of plant-based food produces large amounts of by-products, e.g., pomaces, which are natural sources of biologically active compounds. Therefore, it is utmost importance to find new ways to valorise these valuable natural food materials.

Marination is used to preserve food products, and the use of natural antioxidants in order to improve the quality and nutritional value of fish products is increasing [2,3,4]. Fish and fish products are highly susceptible to microbial spoilage, which reduces shelf life and increases the risk of foodborne infections. Microbiological spoilage is associated with many different microorganisms including *Pseudomonas* spp., yeasts and moulds [5].

There are many ways foodborne pathogens can be transmitted into the food production chain, which can ultimately lead to the contamination of the end products, e.g., ready-to-eat (RTE) foods [6]. Studies by Kramarenko et al. [7] and Koskar et al. [8] have shown a high prevalence and concentration of *Listeria monocytogenes* in certain RTE fish products, which poses serious food safety risks, especially for consumers belonging to risk groups. In addition, RTE fish products have been linked to a prolonged multicountry listeriosis outbreak [9]. According to the European Union Reference Laboratory for *L. monocytogenes* (EURL Lm), RTE foods are susceptible to *L. monocytogenes* contamination, but only those in which the pathogen can survive and/or grow are potential causes of listeriosis [10]. Food business operators need to prove that food safety is ensured throughout the food’s shelf life, and challenge testing is one of the options to verify food safety.

Fruits and berries and their processing residues are known as natural sources of polyphenolic antioxidants, which may possess antimicrobial activity [11,12,13,14]. Some studies have demonstrated the antimicrobial effect of fruit and berry processing by-products in food matrices [15,16]. In the study of Tamkute et al. [17], cranberry pomace extracts were shown to efficiently inhibit meat spoilage and the growth of foodborne pathogens including pathogenic *L. monocytogenes* in pork slurry, burgers and cooked ham.

The antimicrobial activity of fruits and berries is related to flavonoids and organic acids [18]. Of the organic acids, malic, fumaric, succinic, citric and tartaric acids predominate in apples [19]. The most common organic acids in black currants are malic acid, citric acid, quinic acid and ascorbic acid [20]. Citric, oxalic, malic, succinic and tartaric acids are the main organic acids in rhubarb stems [21]. Mostly citric and malic acids are present in tomatoes [22]. It has been found that at the same pH, organic acids are more effective than inorganic acids at bacterial growth inhibition [23]. This has led to their use as preservatives in food production [24].

However, according to our knowledge, studies of the effects of fruit and berry pomace extracts on the growth of bacteria and some other quality indicators in marinated raw fish products are rather scarce. Therefore, this study aimed to evaluate the effect of marinades containing water extracts of apple, black currant, rhubarb or tomato pomaces on the growth dynamics of microorganisms in raw rainbow trout samples within a 22-day study period as well as on the sensory properties of marinated fish. Also, the growth potential of *Listeria monocytogenes* in marinated fish samples was assessed.

## 2. Materials and Methods

### 2.1. Raw Material and Marinade Solutions

Fresh rainbow trout (*Oncorhynchus mykiss*) fillets (Trim B) were purchased from a local fish farm in Jõgeva County, Estonia. The fillets were kept on ice at a temperature of −1…+3 °C in cold storage. The day after the fish were caught, the fillets were cut into 2 × 4 cm pieces, and the cutting area was selected to ensure that the pieces were of equal thickness.

Pomaces obtained from the juicing of apples (*Malus domestica* Borkh.), black currants (*Ribes nigrum* L.), rhubarb (*Rheum rhaponticum* L.) and tomato (*Lycopersicon esculentum* Mill.) were used as raw material for powder preparation at the laboratories of the Polli Horticultural Research Center of Estonian University of Life Sciences. The marinating solutions (Table 1) consisted of aqueous extracts of the apple, black currant, rhubarb or tomato pomaces and additionally contained 3% sugar, 3% salt, 1% acetic and 0.25% citric acid. The content of the phenolic compounds in the pomace powders is given in Figure S1.

Pomace extracts were prepared in a ratio of 1:10 (*w*/*v*), calculated for a solids content of 10%. Mixtures of distilled water and pomace powders were heated in a water bath at 70–80 °C for 30 min.

The marinating solutions (marinades) were bottled in sterile glass bottles, pasteurized at 70–80 °C for 20 min, sealed airtight and cooled. Then, sugar, salt, acetic and citric acid were added to the marinades. The pH of the prepared solutions was measured (Table 1). The marinades were stored in the dark under refrigerated conditions until further use.

### 2.2. Sample Preparation

The fillet pieces were weighed into plastic boxes (one box for one sampling day) and covered with the marinating solution at a ratio of 1:1. A lid was placed on the plastic boxes, and the boxes were stored in a refrigerator at a temperature of 6 ± 1 °C. The fillet pieces were marinated for 22 days, and samples were taken on days 1, 4, 8, 11, 15, 18 and 22. The challenge test of the marinated fish samples was carried out for up to 15 days. All analyses were performed in triplicate.

### 2.3. pH Determination

Homogenates made up of 10 g of sample and 100 mL of distilled water were used to measure the pH values of the samples. A digital HandyLab680 pH meter (SI Analytics GmbH, Mainz, Germany) was used to take the readings at room temperature. The pH meter calibration was regularly checked.

### 2.4. Water Activity (a_w_) Determination

Water activity (a_w_) was determined at 25 °C, using an Aqualab Decagon 3TE water activity meter (Decagon Devices Inc., Pullman, WA, USA) following the manufacturer’s instructions.

The water activity and pH were measured on the same measurement day in parallel.

### 2.5. Enumeration of Microorganisms

For the enumeration of total microorganisms [25], yeasts and moulds [26], presumptive *Pseudomonas* spp. [27] and for the detection [28] and enumeration [29] of *L. monocytogenes* standard methods were followed. Briefly, a Kern KB 2000-2N scale (Kern & SOHN GmbH, Balingen, Germany) was used to weigh 10 g of the material into a sterile Stomacher bag; the sample was then diluted with 90 mL of sterile buffered peptone water (LAB204, Lab M, Lancashire, UK) to obtain an initial 10-fold dilution. Samples were blended for one minute at 230 rpm using a Stomacher™ 400 Circulator (Seward, UK).

Plate Count Agar (PCA, LAB010, Lab M, Lancashire, UK) was used for the enumeration of microorganisms, DRBC Agar (ISO) (LAB217, Lab M, Lancashire, UK) was used for the enumeration of yeasts and moulds, *Pseudomonas* Agar Base (CFC, Biolife, Italiana S.r.l. Viale Monza, Milano, Italia) was used for the enumeration of presumptive *Pseudomonas* spp. and Agar Listeria Ottaviani Agosti (ALOA) was used for the enumeration of *L. monocytogenes* (Biolife, Italiana S.r.l. Viale Monza, Milano, Italia). For the analyses, the surface plating technique was used. For small and large agar plates, 100 µL or 1000 µL of the initial dilution was spread onto the agar surfaces, respectively. Plates for counting total microorganisms were incubated under aerobic conditions at 30 °C for 72 ± 3 h, yeast and mould agar plates (DRBC) were incubated at 25 °C for 5 days, agar plates for *Pseudomonas* spp. (CFC) were incubated at 25 °C for 44 ± 4 h and plates for *Listeria* (ALOA) were incubated at 37 °C for 24–48 h. After incubation, the colonies were enumerated, and the results were presented as log_10_ colony-forming units per gram (cfu/g).

### 2.6. Challenge Test

The suspension used for challenge testing contained a mixture of two *L. monocytogenes* strains (12MOB101LM, genoserotype II and 12MOB102LM, genoserotype IV) originating from the EURL Lm strain collection. Both strains were isolated from fish. The challenge test was carried out in accordance with the technical guidance document (version 4) of the EURL Lm. Samples without pomaces contaminated or not contaminated with *L. monocytogenes* were used as positive and negative controls, respectively.

Three samples for each day of analysis were artificially contaminated with the microbial suspension using a syringe. The volume of the inoculum introduced into the food matrix did not exceed 1% of the whole mass. Different points of the sample were injected with the total of 100 μL of the mixture at an approximate concentration of 100 cfu/g. The inoculated samples were placed separately into sterile cups, and the marinade was added after the inoculation. Samples were stored in an incubator at 6 ± 1 °C and analysed on days 1, 4, 8, 11 and 15. The total microbial count, pH and water activity (a_w_) were also determined on each day of analysis in triplicate. Based on the enumeration of *L. monocytogenes*, the growth potential of the pathogen was determined.

### 2.7. Sensory Evaluation

The sensory properties of the samples were assessed using the hedonic and just-about-right (JAR) scales of the Fizz by Biosystems (version 3.7.3) software (Couternon, France). Twelve panellists from the Chair of Veterinary Biomedicine and Food Hygiene and Chair of Food Science and Technology evaluated the sensory properties of the marinated rainbow trout on day 4 of marination. Pieces of fish from each treatment were presented to the panellists on a tray labelled with a three-digit random number. White bread and warm black tea were provided to neutralize the mouth between the samples.

The panellists were asked to rate the samples for the appearance of the marinade and the flesh, odour, flavour, aftertaste, texture and overall acceptance on a 9-point hedonic scale (1—“dislike extremely” and 9—“like extremely”); intensity levels of saltiness, acidity and consistency were evaluated on a 5-point JAR scale (“0”—just about right). For evaluation, panellists used individual booths.

### 2.8. Statistical Analyses

Microsoft Excel 365 (Microsoft Corporation; Redmond, WA, USA) was used to record the results, and R v4.2.3 was used for statistical analyses [30]. A two-factor analysis of variance was performed to compare *L. monocytogenes* logarithmic counts in samples with different marinated fish samples and on different study days within the challenge test. Tukey’s post hoc test was used for pairwise comparison analysis. Additionally, correlation analysis was performed separately on different samples using the sensory panel evaluation data regarding appearance, smell, taste, juiciness, aftertaste and overall rating. Results were considered statistically significant at *p* < 0.05.

## 3. Results

### 3.1. pH and a_w_ in Marinated Fish

**Figure 1:**
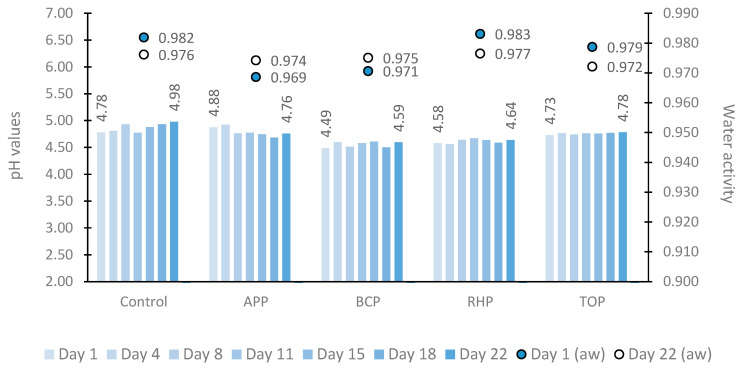
The pH (bar chart) and water activity (point chart) in marinated fish samples on storage days 1, 4, 8, 11, 15, 18 and 22. Abbreviations: APP, apple pomace; BCP, black currant pomace; RHP, rhubarb pomace; and TOP, tomato pomace.

The dynamics of pH and water activity in marinated fish products are shown in Figure 1. The pH values varied greatly between different samples at each time point tested depending on whether or which plant pomace the sample contained in this study. However, throughout the 22-day study period, the pH of the control sample remained higher (4.78 to 4.98) than those of the other tested samples as follows: apple pomace (APP) (4.88 to 4.76) and tomato pomace (TOP) (4.73 to 4.78) samples. The lowest pH values were observed in the fish samples marinated in rhubarb pomace (RHP) (4.58 to 4.64) followed by black currant pomace (BCP) (4.49 to 4.59).

Within the storage period, the lowest and highest water activity values were 0.969 and 0.983, respectively. Throughout the study period, higher water activity values were observed in the control (a decrease from 0.982 to 0.976) and RHP (0.983 to 0.977) samples. Water activity was lower in the APP (an increase from 0.969 to 0.974) and BCP (0.971 to 0.975) samples.

### 3.2. Microorganisms in Marinated Fish

**Figure 2:**
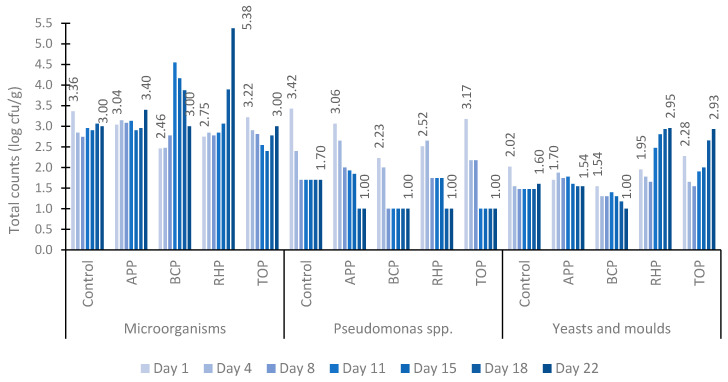
The counts of total microorganisms, *Pseudomonas* spp., yeasts and moulds in marinated fish samples on days 1, 4, 8, 11, 15, 18 and 22. Abbreviations: APP, apple pomace; BCP, black currant pomace; RHP, rhubarb pomace; and TOP, tomato pomace.

The dynamics of the counts of total microorganisms, *Pseudomonas* spp., yeasts and moulds in marinated fish products are presented in Figure 2. In all tested samples, the total number of microorganisms was higher than the counts for *Pseudomonas* spp. and yeasts and moulds during the 22-day study period. Initially, the total number of microorganisms averaged between 2.46 and 3.36 log_10_ cfu/g on the first day and 3.00 and 3.40 log_10_ cfu/g on the last day of the experiment, except for the RHP sample, which contained 5.38 log_10_ cfu/g on day 22. However, the total microbial counts remained at an acceptable level in all tested samples.

In contrast to the increase in the counts of microorganisms, there was a clear decrease in the number of *Pseudomonas* spp. from 3.42 log_10_ cfu/g to 1.00 log_10_ cfu/g in all samples during the 22-day testing period. Similar dynamics of the counts were observed in both the control sample and the samples containing pomaces.

Similarly to those of *Pseudomonas* spp., the numbers of moulds and yeasts were also low in all tested samples throughout the study period remaining between 1.00 and 2.95 log_10_ cfu/g. However, while the APP and BCP samples showed a decrease in the numbers of moulds and yeasts from 1.70 to 1.54 log_10_ cfu/g and 1.54 to 1.00 log_10_ cfu/g, respectively, the total numbers in the RHP and TOP samples increased up to tenfold by the end of the study period.

### 3.3. Challenge Testing

For the control of *L. monocytogenes* in raw material, the standard protocol [28] was followed. All samples were negative for *L. monocytogenes*. The results of the *L*. *monocytogenes* challenge tests in the marinated fish samples are presented in Table 2. The growth potential of *L. monocytogenes* was lower than 0.5 log_10_ cfu/g in all tested samples during the 15-day challenge period. The lowest growth potential (δ = 0.169 log_10_ cfu/g) was found in the control sample followed by the TOP (δ = 0.209 log10 cfu/g) and APP (δ = 0.293 log_10_ cfu/g) samples. Although the RHP sample had a higher growth potential of 0.464 log_10_ cfu/g, it did not exceed 0.5 log_10_ cfu/g. The RHP fish sample had a lower pH (4.58 ± 0.17 to 4.64 ± 0.06) than the other samples (on average 4.769 ± 0.07), but the water activity (average 0.969 ± 0.003) was similar to the other samples (average 0.972 ± 0.003).

The total mesophilic aerobic counts in the challenge tests of the marinated fish samples increased over 15 days in the BCP (from 2.46 to 4.16 log_10_ cfu/g) and RHP (from 2.75 to 2.90 log_10_ cfu/g) samples but decreased less than tenfold in the control, APP and TOP samples. The total microbial counts remained at acceptable levels in all tested samples. In this study, the tolerable value for the total microbial count was 6.0 log10 cfu/g.

The average counts of *L. monocytogenes* on different days and in the tested marinated fish samples are shown in Table 3. The initial counts of *L. monocytogenes* in all tested samples on day 1 of storage were similar, averaging 2.41 ± 0.084 log_10_ cfu/g. Also, on the following days of storage, all samples contained similar numbers of *L. monocytogenes* on average, namely, 2.43 ± 0.414 log_10_ cfu/g on day 4, 2.38 ± 0.148 log_10_ cfu/g on day 8 and 2.38 ± 0.189 log_10_ cfu/g on day 11. However, on the last day of the challenge experiment, significantly lower (*p* < 0.05) average numbers of *L. monocytogenes* (on average 2.17 ± 0.498 log_10_ cfu/g) were detected than on the previous storage days.

Comparing the initial and final results, a tenfold decrease in the average counts of *L. monocytogenes* was found for the BCP samples, followed by the APP and RHP fish samples. Throughout the study period, the lowest average numbers (*p* < 0.05) of *L*. *monocytogenes* were observed in the BCP fish samples (on average 2.35 ± 0.235 log_10_ cfu/g) compared to the control samples.

*L*. *monocytogenes* growth potential dynamics between tested marinated samples on different storage days are shown in Table 4. The highest overall positive growth potential occurred on day 4 both in the APP (δ = 0.293) and in the RHP (δ = 0.464) samples, on day 8 in the TOP (δ = 0.209) samples, on day 11 in the BCP samples (δ = 0.321) and on day 15 in the control samples (δ = 0.169). The growth dynamics of *L. monocytogenes* in the marinated fish samples showed that for the last day of the experiment, the numbers of *L. monocytogenes* remained lower compared to the control. On day 15, the lowest growth potential of *L. monocytogenes* was found for the BCP and APP fish samples.

### 3.4. Sensory Evaluation

**Figure 3:**
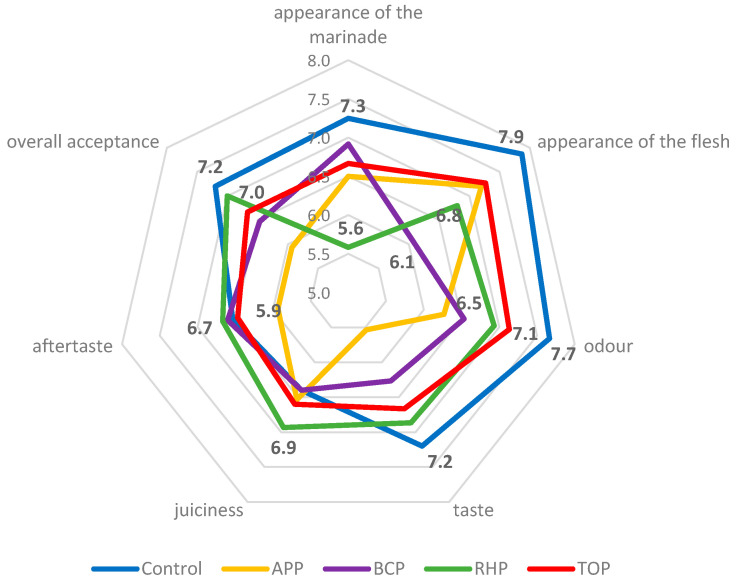
The sensory attributes of the marinated rainbow trout samples. Abbreviations: APP, apple pomace; BCP, black currant pomace; RHP, rhubarb pomace; and TOP, tomato pomace. The bold numbers in the figure are the scores, which are reflected in the results and discussion of the article.

The results of the sensory evaluations are shown in Figure 3. The appearance of the acetic acid marinade as the control was scored with the highest points (7.3), and the marinade with RHP with the lowest (5.6). The appearance of the flesh in the control sample was also scored with the highest points (7.9), but the unusual purple colour of the flesh in the marinade with BCP resulted in this sample scoring the lowest (6.1). The odour, taste and overall acceptance of the fish in the acetic acid marinade were evaluated with the highest points, namely, 7.7, 7.2 and 7.2, respectively. The highest points for juiciness and aftertaste, namely, 6.9 and 6.7, respectively, were given to the fish in the marinade with RHP.

**Figure 4:**
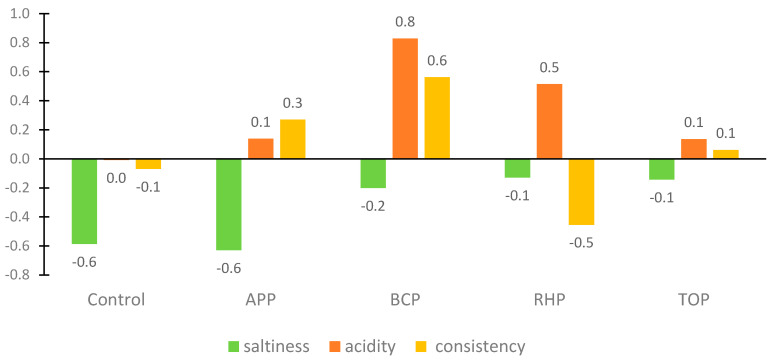
Results of the just-about-right (JAR) evaluation of the marinated rainbow trout samples. Abbreviations: APP, apple pomace; BCP, black currant pomace; RHP, rhubarb pomace; and TOP, tomato pomace.

The appearance, colour, odour and taste of the flesh depended on the pomaces used in the marinade. The APP gave a strong apple smell and taste, which some panellists considered unusual, peculiar and too sweet, while others liked it. The BCP turned the fish an unusual dark purple colour, adding acidity and a peculiar taste that the panellists found surprisingly good. The RHP gave the marinade an orange-pinkish colour, added acidity, made the flesh consistency quite soft and gave the product a delicate sour smell. The appearance of the marinade with the TOP was slightly more orange than the others. The flesh had a soft consistency, was slightly sour and had a good tomato flavour and taste that most panellists liked; however, some did not, and some would have preferred even more salt. Results of the just-about-right (JAR) assessment are shown in Figure 4.

Consistency and taste intensity, such as saltiness and acidity, were assessed on a 5-point JAR rating scale ranging from “too little’’ to “too much”, with “0” indicating “just about right”. The JAR results showed a positive acceptance of the products. However, the JAR scores showed that the panellists were not satisfied with the consistency of the flesh in the RHB marinade sample and would have liked a bit more saltiness and less acidity in the BCP and RHB marinade samples. Correlation analyses showed a statistically significant (*p* < 0.05) positive correlation between the overall rating and the appearance of the fish samples in the marinades with the apple and tomato pomaces.

## 4. Discussion

Food business operators are tasked with producing a safe food product that has a sufficiently long shelf life and is acceptable to the consumer. For the inactivation or inhibition of pathogenic and spoilage microorganisms or the suppression of the chemical deterioration of food, a variety of processing and preservation methods are used including marinating fish [31].

The shelf life of a food product is determined by several food quality and/or food safety factors. Food quality can be affected by organoleptic, (bio)chemical and microbiological changes [32]. Food safety of ready-to-eat foods can be compromised by the presence or growth of *L. monocytogenes* or other pathogenic microorganisms to which food safety criteria are applied according to Commission Regulation (EC) No 2073/2005 on microbiological criteria for foodstuffs. Non-legislative food quality and safety criteria can also be established by the food business operators themselves within the self-control system.

In the present study, the general numbers of microorganisms such as mesophilic microorganisms and yeasts and moulds provide a quantitative assessment of the microbiological quality of the marinated fish products within a defined time period of the durability study. While the number of microorganisms provides a quantitative assessment of the microbiological quality of marinated fish products, the total count of *Pseudomonas* spp. indicates the presence of specific spoilage microbes, and the *L. monocytogenes* count is the food safety indicator in RTE foods. For acidified and marinated fishery products, within the shelf life of the product, the target and tolerance level of the aerobic count can be as high as 3 × 10^5^ and 3 × 10^6^ cfu/g [33]. In the present study, within the determined shelf life, the number of mesophilic aerobic microorganisms remained at acceptable levels in all tested samples including the control. The total numbers of mesophilic aerobic microorganisms in the tested samples remained below 5 log_10_ cfu/g, except for that in the RHP fish samples in which the number was 5.38 log_10_ cfu/g (Figure 2). However, all tested marinated fish products can be characterized as having good microbiological quality up to the “use by” date of 22 days. The same applies to the count of yeasts and moulds, for which the tolerance can be up to 3 × 10^4^ [33], but in the present study, the highest value (2.95 log_10_ cfu/g) was determined for fish in RHP containing marinade (Figure 2). The counts of moulds and yeasts and *Pseudomonas* spp. remained acceptable in all tested product samples. Psychrotrophic *Pseudomonas* spp. can be a dominant bacterial species in skin samples of farmed fish [34]. In the present study, the total count of presumptive *Pseudomonas* spp. indicates the levels of specific spoilage bacteria. However, according to Nixon et al. [35], many *Pseudomonas* species are also known as opportunistic pathogens of humans, animals and farmed fish. A total of 90 *Pseudomonas* strains belonging to 20 species were isolated from aquaculture farm water and fish in a recent study by Duman et al. [36].

The present study showed that marinades enriched with fruit and berry pomaces were able to suppress the growth of *Pseudomonas* bacteria as well as to kill them as compared to the initial concentrations; the number of *Pseudomonas* was decreased by about 2 log_10_ cfu/g (100 times) on the last day (day 22) of the determined shelf life. This can be explained by the fact that *Pseudomonas* bacteria are not acid-tolerant and rarely grow below pH 5.0–6.0 [37]. Membré and Burlot’s [38] study has shown that no growth of *P. marginalis* was detected at 4 °C with 5% salt and a pH of 6. In the present study, the pH of the fish samples at 6 °C was below 5 throughout the 22-day study period, namely, 4.78–4.98 for the control samples and 4.49–4.78 for the other tested fish samples. The decrease in the total counts of *Pseudomonas* spp. is probably due to the acidic environment that prolonged the lag phase of *Pseudomonas* spp. for the entire durability study. Gonçalves et al. [39] also observed a long lag phase in the growth of *P. fluorescens* at more acidic pH values. The relationship between pH and the antibacterial effect of organic acids has been previously demonstrated [40,41]. Organic acids are, at the same pH, more effective than inorganic acids in inhibiting bacterial growth [23]. The mechanism is related to the ability of organic acids in the dissociated state to cross the membrane and then ionize in the bacterial cytoplasm [40]. This may cause inhibition of bacterial cell growth because of the lowered cytoplasmic pH, causing osmotic stress, and toxicity of organic acid anions [23,24,41].

Similar to *Pseudomonas* spp., the same phenomenon was observed for *L. monocytogenes* in challenge testing, where the growth potential of the pathogen was decreased in all samples, especially in those enriched with fruit and berry pomaces. Recently, Koskar et al. [42] demonstrated that several fruit and berry pomaces were able to inhibit the growth of microorganisms in raw minced pork samples. However, the same study found that plant powders alone were not able to inhibit the growth of *L. monocytogenes* in minced meat samples.

Regulation (EC) No 2073/2005 establishes the food safety criteria for *L. monocytogenes* in RTE foods. The quantitative limit of 100 cfu/g applies to RTE foods, which are not able to support the growth of *L. monocytogenes* or if the food business operator is able to demonstrate to the competent authority that the number of pathogens in the product will not exceed the limit of 100 cfu/g throughout the shelf life of the food. In challenge testing, the growth potential of *L. monocytogenes* below the criterion of 0.5 log_10_ cfu/g indicates whether the RTE food is able to support the growth of this foodborne pathogen or not [10]. In the present study, the growth potential of *L. monocytogenes* in all samples was below the aforementioned criterion. Furthermore, the average numbers were lowest (*p* < 0.05) on the last day (day 15) of challenge testing. This is a very good indicator of food safety because *L. monocytogenes* is known to be resistant to many environmental stressors including refrigeration and modified atmosphere packaging [9]. Therefore, *Listeria* can grow to high numbers in chilled foods with a long shelf life, especially in certain RTE fish and meat products [7,43,44]. Raw RTE fish products belong to the high-risk food category, due to the high prevalence and counts of *L. monocytogenes* found in these products [8]. Previous studies have shown that some plant materials can be effective in the growth inhibition of foodborne pathogens both in vitro and in foods [17,45,46]. Plants that contain phenolic acids, flavonoids, tannins, alkaloids and glycosides as well as their derivates have been found to have antimicrobial properties [47]. Natural preservatives can also be derived from pomaces obtained from juice pressing, which makes using natural additives both economically and environmentally reasonable. Fruit and berry pomaces consist of skins, seeds and stems with a high concentration of phytochemicals that may have good antimicrobial properties, thus able to prolong food spoilage [48]. This is in good agreement with the results of the current study because the strongest overall microbial growth inhibition effect was found in the BCP fish samples which also had the highest content of polyphenols, including anthocyanins (Figure S1).

There are only a few studies that have analysed the antimicrobial effect of marinades with plant-derived ingredients on food. Wine-marinated beef, which contained essential oils of *Juniperus communis* and *Satureja montana*, had antibacterial, including a listericidal effect in a study by Vasilijević et al. [49]. In a study by Testa et al. [50], olive leaf extracts in marinated anchovy fillets showed an inhibitory effect against all tested pathogenic bacteria, including *Listeria*). Urbonavičiūtė et al. [15] showed that herring coated with films containing cranberry pomace and grape seeds inhibited the growth of *L. monocytogenes* and *Pseudomonas aeruginosa* at 4 °C for 18 days. Also, a study by Zavistanaviciute et al. [16] found that the dairy, berry and fruit by-products used in marinades and their combinations can suppress the growth of pathogenic and opportunistic microorganisms to improve the safety and quality characteristics of lamb meat. The low counts of aerobic mesophilic microorganisms and decreased numbers of *L. monocytogenes* found in this study indicate that raw rainbow trout in fruit and berry pomaces containing marinades can be considered microbiologically safe and of high microbiological quality. Therefore, these products may have the potential for industrial food production.

Another important task of this study was to evaluate the effect of the used fruit and berry pomace ingredients on the sensory quality of marinated rainbow trout. Healthier products are preferred, but also sensory characteristics have a particular significance for consumer preference. It is essential that added plant-based ingredients are in agreement with consumers’ preferences and willingness to buy. The 9-point hedonic scale is a widely used method in terms of comparison purposes, particularly when using novel ingredients, in addition to JAR scaling in product development and determining whether attribute intensity is at an optimal level [51,52].

The control samples, followed by the RHP samples showed good overall acceptance with scores of 7.2 and 7.0, respectively, on a 9-point hedonic scale (Figure 3). For the control samples, the good taste of the marinated fish and the soft texture were highlighted; the RHP samples had an interesting pink marinade, good taste and quite soft consistency. The lowest scores (5.9) for overall acceptance were given to the APP samples. The assessors pointed out that these samples were evenly brownish and had an unusual apple taste, which is foreign to fish. Apple pomace is very prone to polyphenol oxidation during fruit processing [53]; therefore, the colour of the pomace powder tends to become brown eventually, and it may affect the colour of the food products to which it is added. The colour and appearance of a product are noticed as the first and very important sensory characteristics for consumers, and these features determine the purchase decision. In the current study, statistical analyses revealed that the overall sensory rating was positively correlated (*p* < 0.05) with the appearance of the fish samples marinated in the APP and TOP solutions. This was not the case for the control and other samples. The appearance of the marinade and flesh were rated high with scores of 7.3 and 7.9, respectively, for the control samples. The assessors least liked the appearance of the RHP marinade (5.6), although the appearance of the flesh was acceptable (6.8). Black currants are known for their intensive dark-purple, almost black, colour, and the marinade with BCP strongly affected the appearance of the flesh, which obtained the lowest score (6.1) compared to the other samples. Black currants contain a high amount of natural pigments (anthocyanins), and the colour stability depends on acid, salt and sugar concentrations in the existing matrix [54]. In the case of BCP, the anthocyanins present the darkest red colour with the lowest pH and blend with an increase in pH levels, thereby again affecting the appearance of the food if applied as an ingredient. The panellists noted the different appearance compared to the other samples and indicated the uniformly purple colour as the main reason for the low score (do not like the beetroot colour); nevertheless, the BCP samples were reported to have a surprisingly good sour berry taste and smell, and the panellists highlighted these samples as being an “interesting” product.

In marinated products, the specific product characteristics are a synergistic effect of salt and acid content. The best or “just about right” result in the JAR assessment for acidity was awarded to the control samples containing citric acid and acetic acid; for saltiness, these samples were indicated to be a little salty, although some assessors commented that saltiness and sourness were in balance; consistency was evaluated as soft and good (−0.1 on JAR scale). In the case of the addition of the RHB, TOP and BCP ingredients, it was observed that their natural sourness affected positively the “saltiness” level being almost “just about right” (−0.1, −0.1 and −0.2, respectively). It may be hypothesized that by adding sour plant-based ingredients to the marinade, there is no need to add as much salt. In the case of consistency, the results differed among the marinades from −0.5 for RHP to 0.6 for BCP being most of the cases close to “just about right”.

It can be summarised that the results of this study present a basis for using these fruit and berry pomace ingredients in marinades for raw trout with fully acceptable sensory and safety characteristics.

## 5. Conclusions

Valorisation of by-products generated from fruit and berry juice production is in good agreement with the concepts of a circular bio-economy and zero waste. The results revealed that the use of apple, black currant, rhubarb and tomato pomaces in marinades for fish products can ensure a sufficiently long microbiological shelf life of the products. The appearance, consistency, colour, odour and taste intensities of the fish marinades depend on the pomaces and salt content used in the marinade but still have acceptable sensory properties to the consumer. Our findings for the *L. monocytogenes* challenge tests showed that the marinated rainbow trout samples with apple, black currant, rhubarb and tomato pomaces can be classified as “ready-to-eat food unable to support the growth of *L. monocytogenes*” according to Commission Regulation (EC) No 2005/2073 and therefore may have good potential for industrial food production.

## Figures and Tables

**Table 1:** Composition and pH of the marinades.

No	Abbreviation	Marinade Composition	pH
1	Control	0.25% citric acid + 1% acetic acid + 3% salt + 3% sugar	2.24
2	APP	Apple pomace + 0.25% citric acid + 1% acetic acid + 3% salt + 3% sugar	2.78
3	BCP	Black currant pomace + 0.25% citric acid + 1% acetic acid + 3% salt + 3% sugar	2.61
4	RHP	Rhubarb pomace + 0.25% citric acid + 1% acetic acid + 3% salt + 3% sugar	2.48
5	TOP	Tomato pomace + 0.25% citric acid + 1% acetic acid + 3% salt + 3% sugar	3.03

Abbreviations: APP, apple pomace; BCP, black currant pomace; RHP, rhubarb pomace; and TOP, tomato pomace.

**Table 2:** Results of the *L*. *monocytogenes* challenge tests in the marinated fish samples.

Marinating Solution	Storage (Day)	pH *	Water Activity (a_w_) *	Total Count (log_10_ cfu/g) *	δ (log_10_ cfu/g) **
Control	1	4.78 ± 0.15	0.982 ± 0.003	3.36 ± 0.03	0.169
	4	4.81 ± 0.00	0.978 ± 0.003	2.85 ± 0.28	
	8	4.93 ± 0.01	0.976 ± 0.004	2.74 ± 0.06	
	11	4.77 ± 0.07	0.976 ± 0.002	2.95 ± 0.00	
	15	4.88 ± 0.04	0.979 ± 0.000	2.90 ± 0.08	
APP	1	4.88 ± 0.17	0.969 ± 0.006	3.04 ± 0.09	0.293
	4	4.92 ± 0.01	0.969 ± 0.003	3.15 ± 0.09	
	8	4.76 ± 0.02	0.970 ± 0.003	3.08 ± 0.10	
	11	4.78 ± 0.02	0.969 ± 0.000	3.13 ± 0.12	
	15	4.75 ± 0.00	0.965 ± 0.003	2.90 ± 0.16	
BCP	1	4.63 ± 0.17	0.969 ± 0.007	2.46 ± 0.09	0.321
	4	4.75 ± 0.24	0.972 ± 0.007	2.48 ± 0.00	
	8	4.64 ± 0.16	0.968 ± 0.000	2.78 ± 0.21	
	11	4.68 ± 0.16	0.967 ± 0.003	4.55 ± 0.09	
	15	4.67 ± 0.11	0.970 ± 0.003	4.16 ± 0.48	
RHP	1	4.58 ± 0.17	0.983 ± 0.003	2.75 ± 0.12	0.464
	4	4.56 ± 0.03	0.973 ± 0.008	2.85 ± 0.40	
	8	4.64 ± 0.08	0.969 ± 0.003	2.78 ± 0.10	
	11	4.67 ± 0.10	0.971 ± 0.003	2.85 ± 0.04	
	15	4.64 ± 0.06	0.966 ± 0.001	2.90 ± 0.08	
TOP	1	4.73 ± 0.08	0.989 ± 0.000	3.22 ± 0.09	0.209
	4	4.77 ± 0.02	0.972 ± 0.003	2.90 ± 0.00	
	8	4.74 ± 0.04	0.978 ± 0.001	2.81 ± 0.14	
	11	4.76 ± 0.07	0.972 ± 0.001	2.54 ± 0.28	
	15	4.76 ± 0.07	0.968 ± 0.000	2.40 ± 0.43	

* Values are the mean (obtained from analyses in triplicate) ± SD (standard deviation). ** A growth potential (δ) of more than 0.5 log_10_ cfu/g indicates a sample that supports the growth of *L. monocytogenes*. Abbreviations: APP, apple pomace; BCP, black currant pomace; RHP, rhubarb pomace; and TOP, tomato pomace.

**Table 3:** Statistically significant differences (*p* < 0.05, Tukey post hoc test) between the *L*. *monocytogenes* challenge test average counts (log_10_ cfu/g) ± standard deviation on different storage days and in the tested marinated fish samples *.

Marinating Solution	Storage Day					Average
	1	4	8	11	15	
Control	2.48 ± 0.051	2.08 ± 0.051	2.29 ± 0.047	2.43 ± 0.023	2.65 ± 0.014	2.39 ± 0.206
	A, ab	C, c	AB, a	A, a	C, b	AC
APP	2.43 ± 0.115	2.72 ± 0.023	2.23 ± 0.110	2.31 ± 0.015	2.08 ± 0.000	2.35 ± 0.235
	A, a	AB, b	A, cd	A, ac	A, d	A
BCP	2.32 ± 0.029	1.90 ± 0.157	2.35 ± 0.125	2.64 ± 0.000	1.30 ± 0.000	2.10 ± 0.496
	A, a	C, b	AB, a	B, c	B, d	B
RHP	2.46 ± 0.098	2.92 ± 0.007	2.47 ± 0.031	2.11 ± 0.095	2.31 ± 0.045	2.45 ± 0.286
	A, a	A, b	BC, a	C, c	D, a	C
TOP	2.37 ± 0.066	2.55 ± 0.043	2.58 ± 0.065	2.42 ± 0.082	2.48 ± 0.091	2.48 ± 0.098
	A, a	B, ab	C, b	A, a	CD, ab	C
Average	2.41 ± 0.084	2.43 ± 0.414	2.38 ± 0.148	2.38 ± 0.189	2.17 ± 0.498	
	a	a	a	a	b	

* Mean counts of distinct marinating solutions without common capital letters in the same column are statistically significantly different from each other on the distinct storage day. Mean counts of distinct storage days without common small letters in the same row are statistically significantly different from each other for the distinct marinating solution. Abbreviations: APP, apple pomace; BCP, black currant pomace; RHP, rhubarb pomace; and TOP, tomato pomace.

**Table 4:** *L. monocytogenes* growth potential dynamics between tested marinated samples on different storage days.

Marinating Solution	Storage Day *				
	1	4	8	11	15
Control	2.48	−0.405	−0.194	−0.053	**0.169**
APP	2.43	**0.293**	−0.201	−0.120	−0.352
BCP	2.32	−0.419	0.030	**0.321**	−1.021
RHP	2.46	**0.464**	0.015	−0.341	−0.143
TOP	2.37	0.179	**0.209**	0.052	0.113

* The first storage day results are presented as the log_10_ cfu/g of *L. monocytogenes*, and the other storage day results are presented as a relative change from day one. The overall growth potential of *L. monocytogenes* in the challenge test according to the European Reference Laboratory for *L. monocytogenes* method is marked in bold. Abbreviations: APP, apple pomace; BCP, black currant pomace; RHP, rhubarb pomace; and TOP, tomato pomace.

## Data Availability

Data are contained within the article.

## Supplementary Materials

The following supporting information can be downloaded at: https://www.mdpi.com/article/10.3390/microorganisms11122960/s1, Figure S1: The content of phenolic compounds.
