# Supplementary material for: The Effect of Fruit and Berry Pomaces on the Growth Dynamics of Microorganisms and Sensory Properties of Marinated Rainbow Trout
Source: Microorganisms. 2023 Dec 11;11(12):2960. doi: 10.3390/microorganisms11122960 (PMC10745767; doi:10.3390/microorganisms11122960)
Supplement: Supplementary file 1 [file microorganisms-11-02960-s001.zip › microorganisms-2768777-supplementary.pdf]

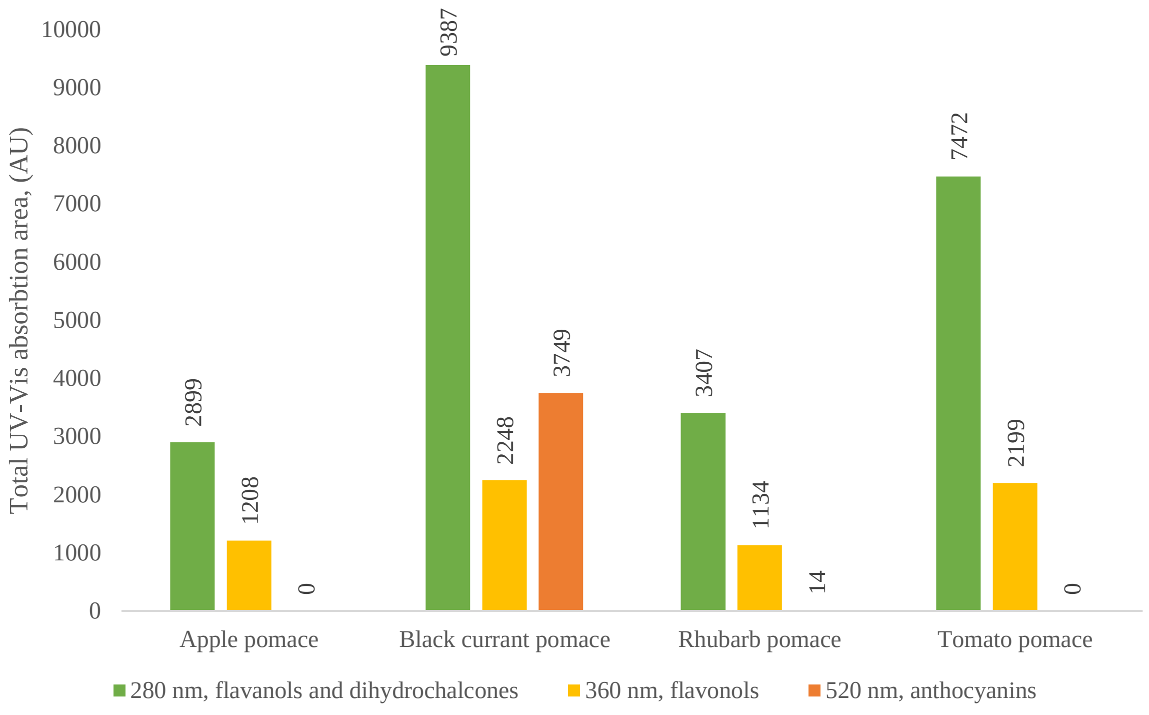

Figure S1. The content of phenolic compounds in 60% ethanol extracts of pomace powders was estimated by HPLC-DAD UV-Vis absorbance at wavelengths of 280, 360 and 520 nm (AU - arbitrary unit). In **apple pomace**, the highest peaks in HPLC chromatograms belonged to chlorogenic acids, catechin mono- and dimers, coumaroylquinic acid, and phloretin derivatives. Compared to other used pomace, the amount of polyphenols in apple pomace was similarly low as in rhubarb pomace. Among studied pomaces **black currant pomace** can be characterized with the highest content of polyphenols, including anthocyanins. The highest peaks in the HPLC chromatogram belonged to phenolic acids, delphinidin, and cyanidin derivatives. **Rhubarb pomace** contained polyphenols, including phenolic acids, catechins, and flavonols. The anthocyanins content was very low. The highest peaks in HPLC chromatogram of **tomato pomace** were tomatoside A, quercetin rutinoside, and rutin pentoside.
